# A Whole-Genome SNP Association Study of NCI60 Cell Line Panel Indicates a Role of Ca^2+^ Signaling in Selenium Resistance

**DOI:** 10.1371/journal.pone.0012601

**Published:** 2010-09-07

**Authors:** Sevtap Savas, Laurent Briollais, Irada Ibrahim-zada, Hamdi Jarjanazi, Yun Hee Choi, Mireia Musquera, Neil Fleshner, Vasundara Venkateswaran, Hilmi Ozcelik

**Affiliations:** 1 Fred A. Litwin Centre for Cancer Genetics, Samuel Lunenfeld Research Institute, Mount Sinai Hospital, Toronto, Ontario, Canada; 2 Department of Pathology and Laboratory Medicine, Mount Sinai Hospital, Toronto, Ontario, Canada; 3 Department of Laboratory Medicine and Pathobiology, University of Toronto, Toronto, Ontario, Canada; 4 Prosserman Centre for Health Research, Mount Sinai Hospital, Toronto, Ontario, Canada; 5 Division of Urology, Sunnybrook Health Sciences Centre, Toronto, Ontario, Canada; 6 Ontario Cancer Institute, Princess Margaret Hospital, Toronto, Ontario, Canada; University of Wuerzburg, Germany

## Abstract

Epidemiological studies have suggested an association between selenium intake and protection from a variety of cancer. Considering this clinical importance of selenium, we aimed to identify the genes associated with resistance to selenium treatment. We have applied a previous methodology developed by our group, which is based on the genetic and pharmacological data publicly available for the NCI60 cancer cell line panel. In short, we have categorized the NCI60 cell lines as selenium resistant and sensitive based on their growth inhibition (GI50) data. Then, we have utilized the Affymetrix 125K SNP chip data available and carried out a genome-wide case-control association study for the selenium sensitive and resistant NCI60 cell lines. Our results showed statistically significant association of four SNPs in 5q33–34, 10q11.2, 10q22.3 and 14q13.1 with selenium resistance. These SNPs were located in introns of the genes encoding for a kinase-scaffolding protein (AKAP6), a membrane protein (SGCD), a channel protein (KCNMA1), and a protein kinase (PRKG1). The knock-down of *KCNMA1* by siRNA showed increased sensitivity to selenium in both LNCaP and PC3 cell lines. Furthermore, SNP-SNP interaction (epistasis) analysis indicated the interactions of the SNPs in *AKAP6* with *SGCD* as well as SNPs in *AKAP6* with *KCNMA1* with each other, assuming additive genetic model. These genes were also all involved in the Ca^2+^ signaling, which has a direct role in induction of apoptosis and induction of apoptosis in tumor cells is consistent with the chemopreventive action of selenium. Once our findings are further validated, this knowledge can be translated into clinics where individuals who can benefit from the chemopreventive characteristics of the selenium supplementation will be easily identified using a simple DNA analysis.

## Introduction

Selenium is an essential dietary trace element with an antioxidant function. It acts as a cofactor for the glutathione peroxidase enzyme and is also incorporated into the selenoproteins that are involved in antioxidant defenses [1], [2]. Selenium is incorporated in mammalian proteins as selenocysteine or selenomethionine, both of which are dietary forms of selenium, although selenomethionine is the major form. Even though at high concentrations it can be toxic to the biological systems, at low concentrations selenium is implicated as a chemopreventive agent in several cancers including breast, prostate, colon, lung and ovarian cancers [1], [2]. It has been particularly studied in relation to prevention of prostate cancer where a direct link between the serum levels of selenium and protection from the prostate cancer was reported [3]–[5]. The chemopreventive action of selenium is attributed to its ability to inhibit cell growth and to induce apoptosis [1].

Given the obvious clinical importance of selenium in prevention of cancer, it is important to understand the characteristics of individuals' response to selenium treatment. Variable response to drugs (such as resistance and toxicity) is an existing issue and can be partly attributed to the wide range of genetic variations among individuals [6]. The likely genetic determinants of variable drug response includes single nucleotide polymorphisms (SNPs), as well as insertions, deletions, and inversions [7] that directly affect gene expression and/or function. Among the genes identified so far in variable drug response are the drug transporters, drug metabolizers, drug receptors, apoptosis-regulating genes and chemokines [6], [8], [9].

To date, identification of drug resistance and toxicity-associated genes has been dependent on candidate gene approaches, which requires prior biological knowledge. Here, we have applied a genome wide association study (GWAS) using genetic (Affymetrix 125K) and pharmacological data from the NCI60 cell line panel [10], with an aim to identify novel genes and genomic regions that are associated with selenium resistance. GWAS results indicated the association of four genes with selenium resistance in the NCI60 cell line panel. RNA interference (RNAi) experiments showed that the down regulation of one of these genes (*KCNMA1*) increased sensitivity to selenium in both selenium sensitive (LNCaP) and resistant (PC3) cell lines.

## Results

### Genetic Analyses

**Figure 1:**
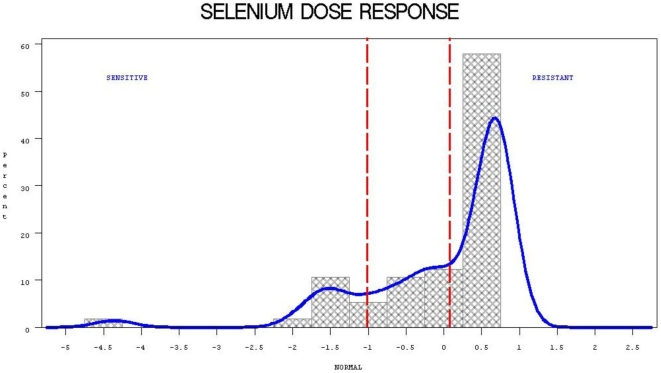
Distribution of NCI60 cell lines with respect to their response to Selenium treatment.

A total of 16 and 30 cell lines were categorized respectively as sensitive and resistant to selenium in the NCI60 cell line panel (**Figure 1** **,** **Table 1**). The single marker whole-genome case-control association test results of 79,622 markers (with minor allele frequencies (mAFs)>2%) have demonstrated the most statistically significant association with resistance to selenium, after conservative Bonferroni correction method (p = 0.0009) and a less conservative Benjamini and Hochberg method (FDR_BH *p* = 0.0009), for a SNP (rs2619641) located within the intronic sequences of K^+^ large conductance Ca^2+^-activated channel, subfamily M alpha member 1 (*KCNMA1*) at10q22. Application of the Bonferroni and/or less conservative FDR_BH correction method has implicated the significance of three other SNPs; SNP rs32076 (p = 0.02) of delta sarcoglycan (*SGCD*) at 5q33, SNP rs10508958 (p = 0.05) of type I (*PRKG1*, also known as *PGK*) at 10q11, and the SNP rs8013938 (p = 0.014) of cGMP-dependent protein kinase A anchoring protein 6 (*AKAP6*) at 14q13 (**Table 2**). Although, *KCNMA1* represented the most likely candidate for further analyses, we have discussed our findings in the context of all four genes.

**Table 1:** NCI60 cell line panel selenium response phenotypes.

	Selenium Resistant Cell Lines	Selenium Sensitive Cell Lines
**1**	786O	A549ATCC
**2**	A498	CAKI1
**3**	ACHN	CCRFCEM
**4**	COLO205	HCT116
**5**	HOP62	HL60
**6**	HT29	HOP92
**7**	K562	IGROV1
**8**	KM12	NCIH226
**9**	LOXIMVI	NCIH23
**10**	M14	NCIH460
**11**	MALME3M	OVCAR4
**12**	MOLT4	SF295
**13**	NCIH322M	SF539
**14**	NCIH522	SNB75
**15**	OVCAR3	U251
**16**	OVCAR5	UO31
**17**	OVCAR8	
**18**	RPMI8226	
**19**	SF268	
**20**	SKMEL2	
**21**	SKMEL28	
**22**	SKMEL5	
**23**	SKOV3	
**24**	SN12C	
**25**	SNB19	
**26**	SR	
**27**	SW620	
**28**	TK10	
**29**	UACC257	
**30**	UACC62	

**Table 2:** Summary of the whole-genome case-control association study for the Selenium resistant and sensitive NCI60 cell line panel.

SNP ID (Affymetrix)	SNP ID (dbSNP)	Chr.	All.	Minor Allele in NCI60	mAF in Resistant Panel N = 30	mAF in Sensitive Panel N = 16	CHISQ	Unadjusted p value	OR	L95	U95	FDR_BH	BONF	Genic Location
2292894	rs32076	5q33	A/G	G	0.018	0.46	24.69	6.74E-07	0.02	0.003	0.19	0.02	0.02	intron 7 of *SGCD*
354475	rs2619641	10q22	A/C	A	0.037	0.57	30.73	2.96E-08	0.03	0.006	0.14	0.0009	0.0009	intron 1 of *KCNMA1*
326494	rs10508958	10q11	G/T	G	0.23	0.77	21.69	3.20E-06	0.09	0.03	0.27	0.05	0.1	intron 4 of *PRKG1*
822011	rs8013938	14q13	G/T	G	0.08	0.58	24.5	7.41E-07	0.06	0.02	0.21	0.014	0.014	intron 1 of *AKAP6*

C**hr**: chromosome; **All**: alleles; **CHISQ**: X^2^; **OR**: odds ratio; **L95**: lower confidence interval; **U95**: upper confidence interval; **FDR_BH**: False discovery rate by Benjamini and Hochberg; **BONF:** Bonferroni method.

The SNP-SNP interaction (epistasis) has been also investigated among the four candidate SNPs using a regression analysis and correction by FDR_BH. As a result, we found statistically significant interaction between the SNPs of *AKAP6* and *SGCD* (*p* = 0.0125) and the SNPs of *AKAP6* and *KCNMA1* (*p* = 0.0125) assuming an additive genetic model. Information for the populations screened and the minor allele frequencies of these SNPs (rs32076 in *SGCD*, rs2619641 in *KCNMA1*, rs10508958 in *PRKG1*, and rs8013938 in *AKAP6*) was available in the dbSNP database [11]. These SNPs were found in the major historic human populations (African, Asian and Caucasian) and were detected in at least 4% of the chromosomes (*not shown*). In order to evaluate the possible involvement of other nearby genes in the observed resistance, we have checked the linkage disequilibrium (LD) structures of these four genes. Our results suggested that the four SNPs identified in this study were located within LD blocks that were part of the *KCNMA1*, *SGCD*, *AKAP6*, and *PRKG1* genes and that did not extend beyond the gene boundaries. Therefore, involvement of other nearby genes in the observed resistance is not likely. An extensive literature search for these 4 SNPs did not reveal any known functional consequences (such as on gene expression or protein function). Thus, the direct biological relationships between these SNPs and resistance to selenium treatment observed in NCI60 cell lines panel remain unknown. However, based on the statistical associations detected, we can hypothesize that the functions of the genes that these SNPs are located in are required for resistance to selenium. Therefore, down regulation of expression of these genes may reverse the resistance to selenium and induce sensitivity to selenium treatment in cells (i.e. cell death would be observed). Thus, to test this hypothesis, we performed RNA interference (RNAi) experiments.

### Functional Analyses

**Figure 2:**
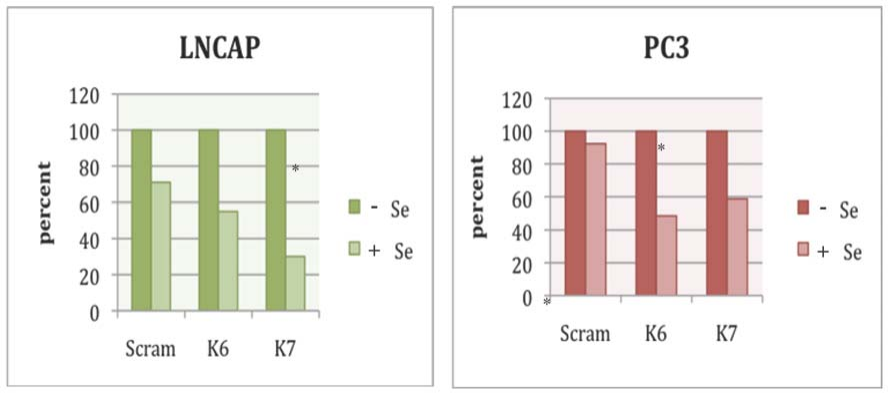
KCNMA1 knock down in LNCaP and PC3 cell lines using siRNA methodology. The percent reduction in response is plotted as a bar graft, the first bar in each set indicating the absence and the second bar representing the presence of selenium treatment of both cell lines. The siRNA experiments are done in triplicates and the average is taken. The (*) indicates the statistically significant (p<0.05) differences in response to selenium treatment.

We have previously reported that LNCaP cell line is relatively much more responsive to selenium treatment when compared to androgen receptor (AR) deficient PC3 cell line [12]. We utilized the RNAi methodology to investigate the selenium response under reduced expression levels of *KCNMA1* (the most significant candidate) in PC3 as well as in LNCaP cell lines. Both cell types were transfected by RNAi probes, and the cell proliferation MTS assays were performed in triplicates. The results were adjusted for the difference between cells with scrambled RNAi, untreated and those treated with selenium. As expected, the treatment of scrambled control cells treated with selenium resulted in 29% reduction in growth of LNCaP cells, whereas there was only a 7.7% reduction in growth observed in PC3 cell line. The siRNA knockdown of *KCNMA1* has shown increased sensitivity to selenium treatment with all the probes where statistical significance was obtained with K7 in LNCaP (p = 0.047) and K6 in PC3 (43.8% difference, p = 0.049) cell lines (**Figure 2**).

To evaluate the portion of the mRNA expression knocked down we have carried out a real-time PCR assay of PC3 cells transfected and untransfected with *KCNMA1* probes 6 and 7, before and after selenium treatment. The treatment with selenium caused a reduction in the expression of *KCNMA1* in PC3 cell lines in comparison to scram cells (untreated with siRNA) (**Table 3**). A further 2-fold reduction in K6 and K7 expression is also observed when compared to the corresponding cells untreated with selenium.

**Table 3:** qRT-PCR results for *KCNMA1* knock down.

	Beta actin			KCNMA1			Ratio KCNMA1/Beta actin
	CT	Gene Copy #	Average	CT	Gene Copy #	Average	
**C**	15.2	2255.1	2176.0	17.5	2691.8	2937.3	1.4
**C**	15.3	2096.8		17.3	3182.7		
**C+SE**	14.1	4546.9	4531.9	16.9	4154.5	4076.9	0.9
**C+SE**	14.1	4516.9		17.0	3999.3		
**Sc**	14.4	3607.1	3756.1	17.0	3909.0	4000.3	1.1
**Sc**	14.3	3905.1		17.0	4091.7		
**Sc+SE**	13.8	5508.5	5490.4	16.9	4154.5	4477.8	0.8
**Sc+SE**	13.8	5472.2		16.8	4801.0		
**K6**	14.6	3331.8	3245.9	18.5	1316.0	1301.1	0.4
**K6**	14.6	3160.1		18.5	1286.3		
**K6+SE**	14.6	3266.3	3277.2	19.4	648.3	673.9	0.2
**K6+SE**	14.6	3288.0		19.3	699.6		
**K7**	14.5	3443.9	3450.1	18.0	1811.8	1811.8	0.5
**K7**	14.6	3456.2		18.1	1819.9		
**K7+SE**	14.1	4607.5	5265.9	18.3	1486.5	1144.4	0.2
**K7+SE**	13.7	5924.3		19.1	802.3		

**C:** control, **Sc:** scram, **SE:** selenium.

## Discussion

Selenium is an essential trace element and a potent regulator of eukaryotic cell growth. Within the cell, it is incorporated as selenocystein into a small group of 25 selenoproteins that are involved in redox regulation of intracellular signaling and antioxidant function [2], [13]. The selenium-containing enzymes glutathione peroxidases are known to be induced by oxidative stress as well [14]. In addition, genetic polymorphisms of selenoproteins have been shown to affect carcinogenesis indirectly by influencing selenium metabolism [15].

Recent epidemiological and animal model studies have demonstrated that selenium may be an effective chemopreventive agent against several human cancers, including colorectal and prostate cancers [3], [16]–[19]. Specially, in the last decade, selenium alone or in combination with other agents have been speculated as an effective chemopreventive agent against prostate cancer, in spite of some controversy [19]–[21]. For example, a recent report on the SELECT trial implicates that Selenium and/or vitamin E did not prevent prostate cancer [21]. However, it is likely that selenium may be a chemopreventive agent in other cancers. Selenium is thought to exert its anticarcinogenic effect through a variety of mechanisms leading to apoptosis triggered by Ca^2+^ signaling, intrinsic mitochondrial pathway, formation of reactive oxygen species, and activation of caspases [2], [15], [22]–[24].

The activity of selenium is strictly dependent on its serum and tissue concentrations; while the lower concentrations induce cell growth, the higher concentrations inhibit growth and induce cell death [25]–[27]. Uguz et al. have also investigated the effects of different selenium concentrations in HL-60 cells, where they have demonstrated that at low concentrations (200 nM) selenium induces a mild endoplasmic reticulum (ER) stress whereas this stress is much more severe at higher concentrations (1 mM) [26]. These studies have demonstrated the dose dependent effects of selenium in mediating cell growth and death via modulating the Ca^2+^ release from the ER [25], [26]. Calcium signaling has been shown to be regulated by the selenoproteins upon selenium supplementation in human endothelial cells [28]. In some studies, selenomethionine was shown to inhibit colon tumor [29] and prostate tumor [30] cell growth at the G_2_/M checkpoint, which was followed by apoptosis. Therefore, these findings indicate a chemopreventive effect of different concentrations of selenium on oxidative stress-induced apoptosis.

In this study, to investigate genetic variations affecting the response to selenium, we utilized an innovative statistics-based method previously reported by us [31]. Our model integrates the analysis of the genome wide genetic data with the response data obtained from the selenium treated NCI60 cell line panel. Our results demonstrated a statistically significant association of the selenium resistance with four SNPs in the intronic regions of four genes; **(a)** ***KCNMA1*** (also known as BK-and Maxi K+ channel), a large conductance, voltage and Ca^2+^-sensitive K+ channel located on the plasma membrane. *KCNMA1* activation has a great impact on the membrane and plays role in muscle tone/contractility and neuronal activity [32] as well as blood pressure regulation [33]; **(b)** ***PRKG1***, a serine-threonine kinase located in the cytoplasm with a key role in nitric oxide/cyclic guanosine monophosphate (cGMP) signaling. *PRKG1* functions directly as a redox sensor directly activated by oxidation in vitro, and in rat cells and tissues [34]. This oxidation-induced activation represents an alternate mechanism for regulation along with the classic activation involving nitric oxide and cGMP; **(c)** ***AKAP6*** **,** a member of the cAMP dependent protein kinase (PKA) anchoring proteins family [35] located on the endoplasmic reticulum/sarcoplasmic reticulum (ER/SR). *AKAP6* is selectively expressed in brain, cardiac and skeletal muscle [36] and it is specifically localized in the SR, therefore, sequestering PKA to this organelle [36]; and **(d)** ***SGCD***, a member of Sarcoglycan family critical in linking cytoskeleton to extracellular matrix. *SGCD* directly stabilizes the link between dystroglycan and dystrophin/utrophin.

**Figure 3:**
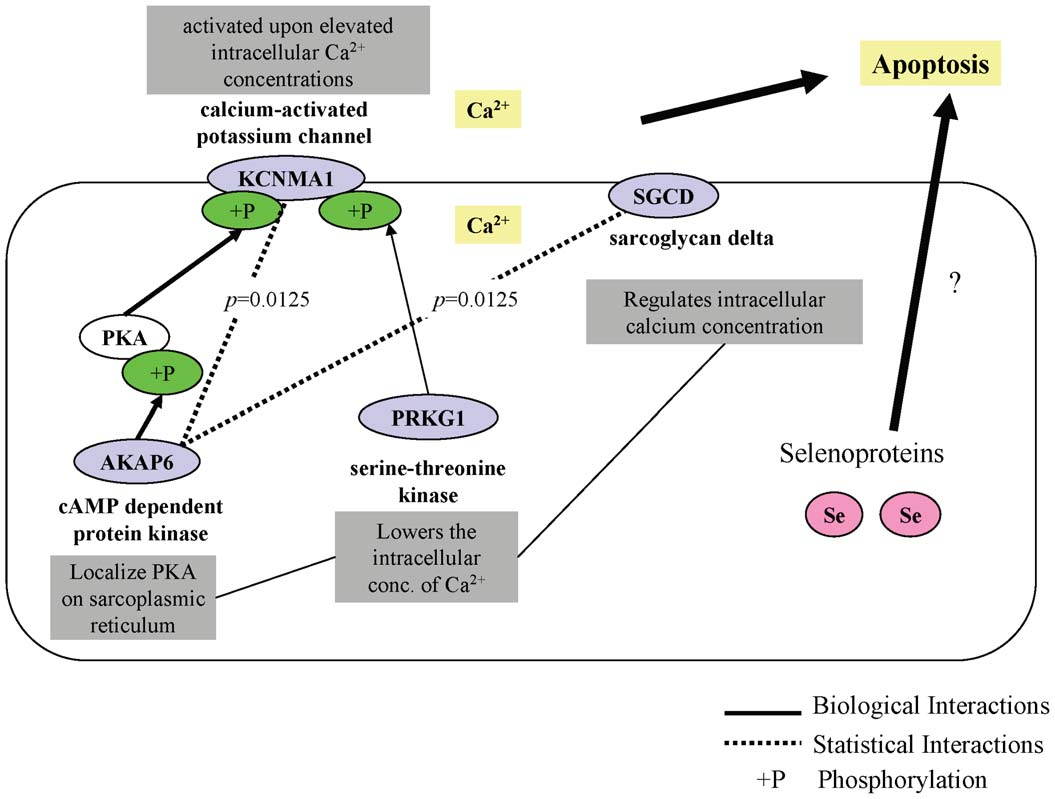
A schematic representation of relationships among the KCNMA1, SGCD, PRKG1, and AKAP6 proteins.

Interestingly all four candidates identified were previously shown to be involved in Ca^2+^ signaling and the regulation of intracellular Ca^2+^ concentrations during cell growth and death. Intracellular Ca^2+^ concentration, a key cellular mechanism regulating cell proliferation and death, is mainly mediated by the plasma membrane associated voltage-gated ion channels as well as the RYR and ITPR channels on the ER. KCNMA1 directly interacts with the alpha subunit of CACNA1C channel [37] leading to Ca2+ influx, opening of KCNMA1 channel, and efflux of the intracellular K+. It also is a key molecule in depolarizing the membrane potential to the resting stage [38]. Interestingly, our second candidate, PRKG1, a serine-threonine kinase, also activates opening of the KCNMA1 channel via phosphorylation [39], [40]. PRKG1 has been shown to lower intracellular concentration of Ca^2+^ in platelets (inhibiting platelet activation), smooth muscle cells (inhibiting contraction), endothelial cells (inhibiting permeability), and cardiac myocytes (depressing contractibility) [41]–[43]. AKAP6, located on the ER/SR, acts as an adapter molecule in co-localization of the PKA, which also phosphorylates and regulates the activity of KCNMA1 [44], [45]. In human dermal fibroblast, PKA is involved in activation of the KCNMA1 by nitric oxide through cGMP [44]. In addition, a recent study reviews the role of AKAP6 in integration of cAMP (i.e. PKA signaling) and Ca^2+^ signaling [46]. The last candidate, SGCD is an essential member of the sarcoglycan complex which was also implicated in regulating intracellular Ca^2+^ concentration [47]. In fact, its deficiency was implicated in abnormal Ca^2+^ regulation [48], while its mutations lead to increased Ca^2+^ permeability and apoptosis in vascular smooth muscle cells of Syrian hamster [49]. Therefore, the functional characteristics of the genes identified in this study are related to each other (**Figure 3**).

These four genes identified in this study also were implicated in human diseases, such as cancer. For example, alteration of KCNMA1 function has been associated with several complex human disorders including cancer. *KCNMA1* expression was found to be higher in breast tumors and brain metastases [50]. *KCNMA1* was also found amplified in 16% of the human prostate cancer [51]. Alterations of *PRKG1* have been implicated in the origin of clonal expansion of primary tumors from breast cancers [52], hepatocellular carcinomas [53] and uterine leiomyomas [54]. Also, according to the COSMIC website, *PRKG1* and *AKAP6* were somatically mutated in some cancers (http://www.sanger.ac.uk/genetics/CGP/cosmic/). SGCD complex, which is involved in proper muscular functioning, has been associated with the limb-girdle muscular dystrophy and cardiomyopathy [47], [55].

Using the RNAi technology, we have investigated the response of PC3 and LNCaP cell lines to selenium treatment in the presence of low expression levels of *KCNMA1*, the most significant candidate gene discovered in the present study. The knock-down of *KCNMA1* by K6 and K7 siRNA probes displayed similar trend of increased sensitivity to selenium treatment of PC3 cell lines. The statistical significance was obtained for K7 probe in relatively sensitive LNCAP cells, whereas the K6 probe was found significant in the relatively more resistant PC3 cell line. The knockdown ratio of *KCNMA1* mRNA expression has validated the reduced expression in cells treated and untreated with selenium. Evaluation of the mRNA expression of *KCNMA1* by qRT-PCR in the significant probe sets (K6 and K7) showed 2-fold decrease in gene expression in the selenium-treated cells. *KCNMA1* is shown to be amplified in primary prostate cancer tumors and PC3 cell line, and siRNA knock-down implicates KCNMA1's critical role in prostate cancer development [51]. The observed variability in response to different probes of *KCNMA1* might be explained by target specificity, and the functional interplay between these and other genes in the cell lines studied.

Finally, given the strong functional relationship among the candidate genes, using statistical methods, we have also investigated the gene-gene interaction (epistasis), demonstrating possible interactions, between the SNPs of *KCNMA1* and *AKAP6* as well as *AKAP6* and *SGCD*, under the additive model. Further studies are warranted to fully investigate the individual and interactive roles of these genes in selenium resistance.

In summary, we have performed a whole-genome case-control study using the already existing NCI60 cell line panel data to identify candidate genes involved in resistance to selenium treatment. Our results identified four genes all of which are involved in calcium signaling, which is in agreement with other studies stating that at least a part of the chemopreventive characteristics of selenium is through its ability to kill tumor cells by inducing apoptosis or by its antioxidant capabilities. However, we also highlight the fact that the combination of genes rather than a single-gene effect may lead to the lack of stronger response in cells, therefore further studies are warranted. Identification of the true genetic basis of the resistant/sensitive phenotypes by further studies will help understanding the molecular basis of selenium resistance. Once confirmed, these findings will find a direct application in clinics where individuals who can benefit from selenium supplementation can be identified using a simple and straightforward DNA test.

## Materials and Methods

### Categorization of Selenium Resistant and Sensitive NCI60 Cell Lines

We have followed a methodology previously described by our group [31], which is based on the publicly available biological and pharmacological data on the NCI60 cell lines [10]. In short, the GI50 data (the amount of the tested compound, in this case selenium methionine, required to inhibit growth of 50% of the cells) were obtained from the Developmental Therapeutics Program (DTP) website (http://dtp.nci.nih.gov/index.html). To determine selenium resistant and sensitive NCI60 cell lines, the log10 of GI50 values at concentration of 10^−4^ M were normalized to obtain a mean of zero and standard deviation of one. Then SAS 9.1 (PROC UNIVARIATE) was applied to estimate the density function of the normalized GI50 values using a normal kernel estimation procedure with an optimal bandwidth estimated at 0.2467 and an asymptotic mean integrated squared error (AMISE) of 0.0206. The density function showed three modes: the NCI60 cell lines with values higher than 0.1 were assigned to the resistant group whereas the cells with values below 0.1 were assigned to the sensitive group (**Figure 1**). A total of 16 and 30 cell lines were defined as sensitive and resistant, respectively (**Table 1**).

### Whole-genome Case-Control Association Study

The Affymetrix 125K SNPs data for the NCI60 cell line panel [56] was downloaded from the DTP website (http://dtp.nci.nih.gov/mtargets/download.html) and was utilized in a whole-genome single SNP case-control association analysis performed by the PLINK software [57] using the standard chi-square test on allelic frequencies. The information related to the populations genotyped for the three SNPs with rs numbers and their minor allele frequencies were retrieved from the dbSNP database build 128 [11]. The PLINK parameters were set to include SNPs that have been genotyped in at least 75% of the cells and had a minimum minor allele frequency (mAF) of 2%. A total of 79,622 markers satisfied these criteria and were used for the association testing. Correction for the multiple testing to decrease the false-positive associations was performed by PLINK using methods such as the False Discovery Rate by Benjamini and Hochberg (FDR_BH) [58]. Results with p values <0.05 were considered significant. The information related to the populations genotyped for the four SNPs and their minor allele frequencies were retrieved from the dbSNP database build 128 [11].

### Linkage Disequilibrium Structures

The genotype data for the European samples (CEU) for the *KCNMA1*, *SGCD*, *AKAP6*, and *PRKG1* genes were retrieved from the International HapMap Consortium database [59]) and analyzed using the Haploview program [60].

### SNP-SNP Interaction Analysis

We used the logistic regression model to analyze two-way interactions among the SNPs that were found individually associated with selenium resistance. This analysis was done using an additive model for each SNP. To test the significance of SNP-SNP interaction, we fit and compare two models, one with the SNP main effects only and the other with the main effects and two-way interaction effect, and calculate the likelihood ratio test and its associated p-value based on ANOVA. The results were corrected for multiple testing using the FDR_BH [58]. This correction accounts only for the number of SNP-SNP interactions performed among the SNPs found individually significant.

### siRNA Transfection of Prostate Cancer Cells

Two established human prostate cancer cell lines, LNCaP (androgen responsive) and PC3 (androgen independent) were obtained from the American Type Culture Collection (Rockville, MD). LNCaP cells was cultured in RPMI 1640 with L-glutamine (Life Technologies, Inc., Grand Island, NY), supplemented with 10% FBS, and 100 IU/ml penicillin and 100 µg/ml streptomycin. PC3 cells were cultured in DMEM/F12 medium with 10% FBS and antibiotics. All of the cell types were maintained at 37°C in a humidified atmosphere of 5% CO2 in air. LNCaP and PC3 cells cultured in medium were either transfected with siRNA (QT00065660 *SGCD* primers; QT00024157 *KCNMA1* primers) or remained untransfected, and treated with selenium or untreated in triplicates. A total of 12.5 ng siRNA in 1–3 µl of siRNA in suspension Buffer/RNase-free water was spotted into a single well of a 96-well plate. Diluted HiPerFect Transfection Reagent was added to the pre-spotted siRNA (0.75 µl of HiPerFect Transfection Reagent to 24.25 µl of culture medium without serum) and incubated for 5–10 min at room temperature (15–25°C) to allow formation of transfection complexes. Following these 5000 cells in 175 µl of an appropriate culture medium (containing serum and antibiotics) was seeded into the well, on top of the siRNA–HiPerFect Reagent transfection complexes. Cells were incubated with the transfection complexes under their normal growth conditions for 6–24 hours. Cells were treated with selenium (150 µM) or vehicle alone and proliferation was monitored after 72 h by the MTS assay as described previously [12]. All experiments were carried out in triplicates at the same time and under similar culture conditions. The difference between the cells transfected with scram versus siRNA probes was tested by paired t test after adjustment for the response difference of scram.

### Real Time Quantitative PCR (qRT-PCR)

PC3 cells cultured in medium were either transfected with siRNA or untransfected, and treated with selenium or untreated. After 72 hrs of incubation, about 3×10^6^ cells were harvested from respective plates and used for RNA extraction. RNA extraction was done using RNeasy mini kit (Qiagen Inc, Canada) according to the manufacturer's protocol. Contaminating chromosomal DNA was digested with RNAse free DNAse (RNAse free DNAse kit, Qiagen inc, Canada) following the optional step during the RNA extraction protocol. Further RNA was analyzed both qualitatively and quantitatively by measuring OD 260/280.

A one step real time PCR using the *KCNMA1* primers (Qiagen) was performed in special 96-well TaqMan optical reaction plate format on a ABI Prism 7000 sequence detection system (Applied Biosystems, Foster city, CA, USA). Beta-actin was used as the endogenous control gene. Each 50 µl reaction contained Qiagen SYBR green master mix, gene specific forward and reverse primer and probes for target and control gene (Qiagen Inc, Canada). To obtain PCR conditions with reduced variability, premixes containing primers and probes and PCR master mix except template RNA were aliquoted into 96-well optical reaction plates. Standard, unknown samples and no template control (NTC) were added in a volume of 5 µl. Serial dilutions of standard ranging from 1∶5 dilutions (5 fold dilutions) were set up for beta actin control and *KCNMA1* target in separate duplicate wells. Thermal cycling parameters included, reverse transcription 30 min 50°C, PCR activation step 15 min 95°C, followed by 40 cycles of 15 seconds at 94°C, 30 s at 55°C and 30s at 72°C. The number of target gene copies was extrapolated from a standard curve equation generated with serial dilutions of a known amount of standard RNA. The normalization of samples was performed by dividing the number of copies of *KCNMA1* gene by the number of copies of beta actin. All further PCR for *KCNMA1* mRNA quantification were performed using standard RNA dilution curves. The intra assay variability was <3%.
